# Development of Emotional Intelligence Through an Intervention Program in Primary School Students

**DOI:** 10.3390/ejihpe15120247

**Published:** 2025-12-02

**Authors:** David Molero, Gemma Sánchez-Narváez, Raquel Jiménez-delaTorre, Óscar Gavín-Chocano

**Affiliations:** 1 Department of Education, University of Jaén, Campus las Lagunillas s/n, 23071 Jaén, Spain; gsn00002@red.ujaen.es (G.S.-N.); rjt00004@red.ujaen.es (R.J.-d.); 2 Research Group: Lifelong Education, Neuropedagogical Integration, Department of Education, University of Jaén, 23071 Jaén, Spain

**Keywords:** emotion, emotional intelligence, emotional development, primary education, well-being

## Abstract

Background: This research describes the influence of emotions on primary school students in a public school in Andalusia, southern Spain. The objectives include determining self-reported emotional intelligence scores, establishing correlations between the instruments, analysing differences according to gender, and studying the influence of an intervention programme on students’ self-reported emotional intelligence scores, by means of pre-test and post-test. Methods: The study design combines descriptive and correlational approaches through a quasi-experimental study. The sample consists of 149 primary education students. A socio-demographic questionnaire was used to assess self-reported Emotional Intelligence, short version in Spanish of the Emotional Education Questionnaire and subscale of the WLEIS-S (version in Spanish) instrument to assess emotional regulation. Results: The experimental group in this study showed a significant increase (pre-test vs. post-test) in emotional intelligence scores following the intervention program (*p* < 0.05, *p* < 0.01) with a large effect size (*r* > 0.5). Conclusions: The findings suggest that emotional education and EI among primary school students have a significant and positive influence on their emotional well-being.

## 1. Introduction

Recognizing and managing emotions is a skill that can be complex, especially for children in primary education, as they are still developing their own personalities. Their current emotional state will determine how they perceive their experiences at that moment. Furthermore, for educators, adequately, positively, and comprehensively understanding the emotions of their students can also be a complex task (Gavín-Chocano et al., 2025). Therefore, it is crucial to foster an environment where the student feels understood and supported during the process of emotional development (Casino-García et al., 2024; Eriksen & Bru, 2022). Emotional development and Emotional Intelligence (EI) are relevant in all aspects of life, including personal relationships, academic performance, and mental and emotional health (Mansfield et al., 2021; Pérez-Escoda et al., 2021).

In recent years, the necessity of implementing educational interventions to promote and foster the mental health and well-being of children and adolescents has become evident (Alarcón-Espinoza et al., 2025; Eriksen & Bru, 2022; Gomis et al., 2025; Patafio et al., 2021). Furthermore, the benefits of emotional education have been demonstrated in relation to the development of attitudes toward oneself and others, thus positively influencing the school and family environment (Molero et al., 2022).

EI has been positioned as a necessary mechanism underlying the identification, management, and use of emotional information in adaptive processes, which can promote positive behaviors (Salovey et al., 1995). This concept was theoretically developed in 1990 as a personal ability to adaptively perceive, understand, and regulate one’s own and others’ emotions (Mayer et al., 2016; Salovey & Mayer, 1990). EI is considered a multidimensional construct that relates to parallel cognitive and emotional activity. This fact has led to the emergence of different models to explain EI and guide its connection to people’s performance in daily activities across various contexts (Molero et al., 2022), as well as models that help evaluate the types of emotional and intellectual qualities people possess (Extremera et al., 2020).

The concept of EI was first introduced in an article by Salovey and Mayer (1990). However, the conceptualization of the EI construct is a matter that requires consensus among researchers. Petrides and Furnham (2001) distinguish between two different constructs of EI: EI as a personality trait, on the one hand, and EI as an ability, on the other. EI as an ability has been conceptualized as the capacity to perceive, assimilate, understand, and regulate one’s own and others’ emotions, promoting emotional and intellectual growth. EI as an ability should be measured using maximum performance tests, whereas trait EI would refer to a constellation of behavioral dispositions and self-perceptions concerning one’s capacities to recognize, process, and use emotionally charged information (Petrides et al., 2004).

One of the most productive fields of research in EI is primarily focused on providing evidence of its relationship with psychological well-being and life satisfaction (Biswas-Diener, 2022; Eriksen & Bru, 2022; Sanz-Junoy et al., 2023), which refers to the state of an individual where both objective and subjective needs are met (Kahn & Juster, 2002).

This implies that life satisfaction plays a significant role in the lives of teachers (Gavín-Chocano et al., 2025) and is influenced by various factors such as employment status, age, and health, among others (Casino-García et al., 2024; Prémusz et al., 2023). While different psychological approaches to this concept exist, subjective well-being is perhaps the one that has garnered the most interest in social psychology in recent years (Kelley et al., 2022).

Several studies have found the existence of significant gender differences in EI ratings in school settings, favoring girls (Molero et al., 2010; Prada-Belascoaín & López-Larrosa, 2016; Sanmartín-López et al., 2018). These differences become more pronounced in adolescence, particularly in emotional perception and stress management, with girls excelling in the perception and understanding of emotions and in empathy, while boys tend to show greater adaptability and stress management skills, especially in middle and late adolescence (Cabello et al., 2016; Candeias, 2025). Regarding age, studies suggest that emotional intelligence tends to increase with age, resulting from cognitive maturation and social experiences (Candeias, 2025; Singh & Thapa, 2023).

### Objectives and Hypotheses

After conducting a general literature review on the subject matter, the following objectives were established for this study: (*a*) To determine the self-reported emotional intelligence scores of the student sample, differentiating them according to the dimensions of the data collection instruments used. (*b*) To establish the existence of statistically significant correlations between the dimensions of the instruments considered. (*c*) To analyze significant differences based on gender (boys vs. girls) and by grade level (5th vs. 6th). (*d*) To analyze the influence of an emotional education intervention program on students’ emotional intelligence scores, establishing the existence of statistically significant differences between the scores obtained before the program’s implementation (pre-test) and those obtained at its conclusion (post-test).

To facilitate the verification of the objectives and their empirical validation, we propose the following hypotheses, around which we will present the research results, verifying their fulfillment:

**Hypothesis** **1** **(H1).** *Statistically significant correlations exist between the dimensions of the instruments employed (Emotional Awareness and Control, Self-esteem, Social-Emotional Skills, Life Skills, and Emotional Regulation)*.

**Hypothesis** **2** **(H2).** *There are significant differences in emotional intelligence based on students’ gender and by grade level*.

**Hypothesis** **3** **(H3).** *Differences exist in emotional intelligence scores after the implementation of an intervention program based on students’ emotions*.

## 2. Materials and Methods

Our proposal is based on a quantitative quasi-experimental study, which will combine descriptive and relational methods to establish the existence of significant differences in the variables under study among the Primary Education students who constitute the sample. The descriptive study will use the total number of participants (5th and 6th course of primary education), and the relational part of the study will employ a quasi-experimental design with the subsample of 6th-grade students. After the first data collection moment (pre-test), these students participated in an intervention program for the improvement of emotional education (Renom-Plana et al., 2008), and after its completion, these subjects once again responded to the data collection instruments (post-test). The interval between the evaluation pre-test and post-test was five months.

### 2.1. Participants

We utilized a non-probability, convenience sampling method. The sample comprised 149 primary school students from a single educational institution in a city in southeast of the Andalusia region (Spain). Regarding gender, 51.68% (*n* = 77) were boys and 48.32% *(n* = 72) were girls, 37 boys and 32 girls participated in the 5th grade, while the 6th grade included 40 boys and 33 girls.

The average age of the participants was 10.7 years (±0.58), with ages ranging from 10 to 12 years. In terms of their educational level, 74 (49.7%) were in the 5th grade of primary school, and 75 (50.3%) were in the 6th grade.

As for the statistical power analysis, an estimate was made to achieve a power of 80%, assuming a confidence interval of 95% and a moderate effect size (Hedges’s *g* = 0.5), with an estimate of 106 subjects. The number of study participants exceeded the number of subjects estimated in this calculation (*n* = 149).

### 2.2. Instruments

#### 2.2.1. Sociodemographic Questionnaire

In addition to the information obtained from the instruments used to assess the emotional intelligence of the students in the sample, data on the sociodemographic variables of gender, age in years, and course-group were collected.

#### 2.2.2. CEE-R (Spanish Version of the Emotional Education Questionnaire)

To evaluate self-reported emotional intelligence, the reduced Spanish version of the Emotional Education Questionnaire (CEE-R) was used (Álvarez et al., 2011). This questionnaire consists of 20 items with a four-point Likert scale (1: Never, 2: Sometimes, 3: Often, 4: Always). It is composed of four dimensions, each with 5 items: Emotional Awareness and Control, Self-Esteem, Socio-emotional Skills, and Life Skills and Subjective Well-being. The reliability of the scores obtained for each of the dimensions was as follows: Emotional Awareness and Control: Cronbach’s alpha = 0.72 and McDonald’s omega = 0.74. Self-Esteem: Cronbach’s alpha = 0.82 and McDonald’s omega = 0.83. Socio-emotional Skills: Cronbach’s alpha = 0.65 and McDonald’s omega = 0.67. Life Skills and Subjective Well-being: Cronbach’s alpha = 0.68 and McDonald’s omega = 0.69.

#### 2.2.3. WLEIS-S

As the previous instrument does not assess emotional regulation, we will use a subscale from the Wong-Law Emotional Intelligence Scale-Spanish (WLEIS-S) instrument (Extremera et al., 2019), specifically the subscale that evaluates Emotional Regulation. It consists of 4 items with a seven-point Likert-type scale (1: Never to 7: Always). The reliability of the scores obtained for this emotional regulation subscale is as follows: Cronbach’s alpha 0.80 and McDonald’s omega 0.81.

### 2.3. Procedure

Ethical specifications stipulated in national and international regulations for research involving human subjects were respected, including the completion of informed consent and the guarantee of confidentiality and anonymity of the data obtained. Participation was voluntary and in accordance with the Declaration of Helsinki. The questionnaires were administered individually in the students’ classrooms, using paper and pencil format, accompanied by the class teacher. The activities were integrated into the classroom and school curriculum and were facilitated by the teachers of each group, accompanied by the corresponding author of this paper. For this reason, families were informed of the process. The approximate response time was 10–15 min. Data collection took place between January and June 2024. This research was approved by the Human Research Ethics Committee of the University of Jaén (Spain), with the identification code: protocol code JUL.23/5.LINEA and approval date 21 July 2023.

### 2.4. Intervention Program

In the initial phase of the study, the results obtained from the application of data collection instruments were. The participants were fifth and sixth-grade primary school students from the school where the research was conducted (*n* = 149). Descriptive statistics were obtained, and reliability was analyzed using the alpha and omega coefficients.

Section two details a quasi-experimental study conducted exclusively with sixth-grade students, specifically with a subsample of 75 students divided into three groups or classes (6th A, 6th B, 6th C), each with 25 students. One experimental group (6th A) and two control groups (6th B: Control Group 1, and 6th C: Control Group 1) were established. The 6th-grade students were selected because at this level in Andalusia (Spain), a subject called Values Education exists, which is an ideal context for developing activities such as those described. Consequently, only 6th-grade students participated in the quasi-experimental study.

After the initial measurement in January 2024 (pre-test), students from all three 6th-grade groups participated in the Emotional Education Program for the third cycle of Primary Education by Renom-Plana et al. (2008), which is based on the five dimensions of Emotional Education from the pentagonal model (Bisquerra & Pérez-Escoda, 2007. Specifically, the program focused on Emotional Awareness, Emotional Regulation, Self-esteem, Socioemotional Skills, and Life Skills.

The program’s objectives are based on the Primary Education curriculum of the Spanish educational system, which includes the acceptance of one’s own identity; progress in the acquisition of personal habits; showing participation and solidarity; respecting social, moral, and ethical values, both one’s own and those of others; resolving everyday life situations; and communicating through oral, corporal, and visual means of expression.

A total of 10 sessions of the aforementioned Emotional Education Intervention Program were developed (Renom-Plana et al., 2008), with two sessions dedicated to each of the five dimensions of the pentagonal model used. The content developed in each of the sessions used in this intervention program is detailed below: (1) Emotional Awareness: Session 1 “What I Feel,” Session 2 “The Week’s Emotions”; (2) Emotional Regulation: Session 3 “How Do I Act,” Session 4 “News and Emotions”; (3) Self-Esteem: Session 5 “Something Speaks Inside Me,” Session 6 “The Person I Admire”; (4) Socio-emotional Skills: Session 7 “Feeling and Communicating,” Session 8 “Do We All Understand the Same Thing?”; (5) Life Skills: Session 9 “What I Like,” Session 10 “Simulating Life”.

The intervention program was conducted from January to June 2024. A total of 10 sessions were held (two sessions per month over the five-month duration), which corresponds to one session every 15 days. All intervention program sessions were conducted in the usual classrooms of the three groups, which were air-conditioned using an air-source heat pump (ASHP).

Upon completion of the intervention, at the end of the third academic term (June 2024), the information-gathering instruments (CEE-R and WLEIS-S) were reapplied to assess the five components of the pentagonal Emotional Education model followed in the intervention program (post-test measure). The purpose was to determine whether there were statistically significant differences between the pretest and posttest measures in the EI dimensions of the 6th-grade students. Previous studies (Boobani et al., 2025) have confirmed that intervention programs based on EI and behaviors affect the emotions of schoolchildren

### 2.5. Data Analysis

The data were analyzed for the assumption of normality, and it was found that they followed a non-normal distribution, so non-parametric tests were employed. Descriptive statistics were obtained, and reliability was analyzed using the alpha and omega coefficients.

The non-parametric analyses performed included a correlation study (Spearman’s *Rho*), and various tests for mean differences: Mann–Whitney *U* test (independent samples) and Wilcoxon signed-rank tests for paired samples; to examine differences between pretest and post-test scores for paired samples. The statistical effect size obtained for the tests performed is reported (Hedges’ g or *r*), along with all other requirements for the presentation of results. Statistical power was estimated using the G*Power software package (version 3.1.9.6). All analyses were conducted using Jamovi software (version 2.7.6).

## 3. Results

We present the differentiated results based on each of the objectives and hypotheses considered in our study.

### 3.1. Descriptive and Relational Study

In order to address the hypotheses, we first present the results for each dimension, disaggregated by educational level (5th and 6th grade) and for the total sample (See Table 1). As can be seen from a descriptive comparison of the results (a differential approach will be used later to determine if the differences are statistically significant), sixth-grade students (6th) have higher scores in self-esteem, socio-emotional skills, and life skills. Regarding the dimensions of emotional knowledge and emotional regulation, it is the students from the lower grade (5th) who obtain higher ratings.

In Table 2, the results of the Spearman’s *Rho* correlation conducted between the dimensions of the instruments are presented, providing an answer to Hypothesis 1 (H1) by finding statistically significant correlations between the factors of the scales used. As can be seen, the correlations between the dimensions are statistically significant in all cases (*p* < 0.05, *p* < 0.01, *p* < 0.001), with the exception of the correlations between the dimensions Emotional Awareness and Control and Social-Emotional Skills (Rho_147_ = −0.062, *p* = 0.456), and between Emotional Awareness and Control and Life Skills (Rho_147_ = −0.06, *p* = 0.450).

In reference to the second hypothesis (H2), we analyzed for the existence of statistically significant differences based on gender (boys vs. girls) and grade level (5th vs. 6th). The results from the Mann–Whitney *U*-tests are presented in the following tables (See Table 3 and Table 4).

The descriptive statistics in Table 3 show that, in general, girls scored higher than boys, except for Self-esteem, where boys scored higher (Boys’ Mean = 15.19 vs. Girls’ Mean = 14.83). As can be seen (see Table 3), no significant differences were observed based on gender (boys vs. girls), with the effect size being small for all dimensions analyzed (Hedges’ *g* < 0.2).

Regarding the grade level (5th vs. 6th), the results from the Mann–Whitney *U* test (See Table 4) indicate that, overall, 6th-grade students have a higher score than 5th-grade students. The exceptions are the Emotional Awareness and Control dimension, where 5th-grade students scored higher (5th-grade *Mean* = 9.03 vs. 6th-grade *Mean* = 8.42), and the Emotional Regulation dimension, where both grades had a similar score in both the mean and median (5th-grade *Mean* = 15.23 vs. 6th-grade *Mean* = 15.14; 5th-grade *Median* = 15.00 vs. 6th-grade *Median* = 15.00).

Significant differences based on grade level (5th-grade vs. 6th-grade) were only found in the Social-Emotional Skills dimension, where the scores of 6th-grade students (*Md* = 15, *Range* = 12) were significantly higher than those of 5th-grade students (*Md* = 13, *Range* = 14), (*p* = 0.012, Hedges’ *g* = 0.23), with a small effect size.

### 3.2. Quasi-Experimental Study

The third hypothesis (H3) posited the existence of differences in EI assessments before and after the implementation of an intervention program based on students’ emotions. This was done by analyzing the presence of statistically significant differences between the scores obtained prior to the program’s development (pre-test) and those obtained upon its completion (post-test).

To address this hypothesis, we will only use the subsample of 6th-grade students (*n* = 75), as they are the ones who participated in the intervention program for the development of emotional education within the context of the values education course framed at this educational level. These comparisons were performed separately for each course, as each has its own specificities and peculiarities. We will provide a comparison of the scores obtained in each of the groups between the pre-test and post-test scores, using the Wilcoxon signed-rank test (See Table 5), a non-parametric statistical test used to compare two related groups or samples and determine if there are significant differences between them.

For the Experimental Group, significant differences were observed across all dimensions between pre-test and post-test scores (*p* < 0.05 or *p* < 0.01), with the exception of the Self-esteem dimension. Although there was an increase in the mean score for this dimension, it was not statistically significant. It is noteworthy that the effect size for this group, across all dimensions with significant differences, was either large (Emotional Awareness and Control *r* effect = −0.49, Social-Emotional Skills *r* effect = −0.51; Life Skills *r* effect = −0.64; Emotional Regulation; *r* effect = −0.72). This suggests that the changes in the scores can likely be attributed to the intervention program performed for this group.

In the Control Group 1 (see Table 5), significant differences were observed between the pre-test and post-test in only one dimension: Emotional Awareness and Control (W de Wilcoxon = 33.0, *p* = 0.04; *r* effect = −0.5686). This difference was favorable to the second measurement time (post-test), with a large effect size. It is therefore evident that this group, having received no intervention, did not significantly modify their scores in the studied variables.

Thirdly, we present the results for the Control Group 2. Significant differences were obtained between the pre-test and post-test in this group in three dimensions: Self-esteem (*p* = 0.015, *r* effect = 0.60), Social-Emotional Skills (*p* = 0.033, *r* effect = 0.50), and Life Skills (*p* = 0.009, *r* effect = 0.71). The effect size in all of these was large (*r* effect > 0.50). This finding is notable, as the significant differences obtained for this group were in favor of the pre-test, a result that requires deep reflection and an analysis of what might have occurred in their academic context. What is clear is that non-participation in the intervention program may have influenced the obtainment of these values.

## 4. Discussion

Regarding Hypothesis 1 (H1), the evidence follows similar trends to those reported in other studies (significant correlations exist among the factors of the instruments used). The results obtained from our research on self-reported EI scores of elementary school students show a similar trend to the findings of Vargas-Salvador (2018), who also studied emotions using the same instruments as our study. Concerning H2, no significant differences were found based on gender, and only in one factor based on the course level. In all cases, girls scored higher than boys, although this difference was not statistically significant based on the gender variable. Other studies, however, did find significant gender differences (Molero et al., 2010) in favor of women in a context similar to our proposal, but with different evaluation instruments. In the same vein, other studies (Prada-Belascoaín & López-Larrosa, 2016) also conducted pre-test and post-test comparisons after applying the same emotional intelligence improvement program for elementary school children as the one used in our research.

Regarding the third hypothesis (H3), differences exist in emotional intelligence scores after the implementation of an intervention program based on students’ emotions); the program’s effectiveness in improving emotional development among sixth-grade students aligns with similar studies that provide evidence of the effects of socioemotional programs at this age (Boobani et al., 2025; Corcoran et al., 2018; Echeverría et al., 2020; Gomis et al., 2025). These programs enhance life skills, well-being, overall emotional competence (Boobani et al., 2024; Corcoran et al., 2018; Filella et al., 2014; Taylor et al., 2017), and emotional regulation (Filella et al., 2014; Gomis et al., 2025).

Furthermore, it is noteworthy that the results of our research show significant differences among students depending on whether they participated in an emotional improvement intervention program. A comparison of the pre-test and post-test emotional scores revealed statistically significant improvements in the experimental group that participated in the program. These results are consistent with the findings of other similar studies (Echeverría et al., 2020; Gomis et al., 2025; Pérez-Escoda et al., 2021).

Including emotional competencies brings substantial benefits, enhancing the school environment (Pérez-Guevara & Puentes-Suaréz, 2022) and helping students manage academic and personal challenges (Gomis et al., 2025).

## 5. Conclusions

Based on the evidence obtained, we can conclude that emotional education and EI in primary school students have a significant and highly relevant impact on their emotional well-being, academic performance, and ability to cope with complex situations. This includes their capacity to respond with appropriate behavior to a problem and to establish healthy, positive relationships. Therefore, it is essential for teachers, families, and the students’ immediate environment to actively promote the development of these skills in children from an early age, preparing them to face the emotional and social challenges they will encounter throughout their lives (Eriksen & Bru, 2022; Gomis et al., 2025). This will also enable them to assimilate and comprehend the feelings and emotions that these unexpected challenges will provoke.

According to Pérez-Escoda et al. (2013), emotional awareness—which has been advocated throughout the theoretical framework of this master’s thesis—is the capacity to be conscious of one’s own emotions, including the ability to understand the emotional climate of a specific environment. Emotional regulation is understood as the ability to manage these emotions correctly. Teachers must consider their own feelings and emotions, as well as those of others, thereby fostering empathy in their students (Casino-García et al., 2024; Gavín-Chocano et al., 2025).

After conducting this research and corroborating our findings with those from other studies, we conclude that the emotional development of children becomes more complex as they grow (Alarcón-Espinoza et al., 2025). The transition to adolescence and puberty, along with the shame and unfamiliarity of new feelings and emotions, can make students feel confused about how to act appropriately during this process. During these years, students learn to experience different emotions simultaneously, acquiring the skill to understand affective ambivalence and emotional regulation (Thümmler et al., 2022).

### 5.1. Limitations

Our research relied on the direct collaboration of teachers from the participating courses and the school, while ensuring the anonymity of students in both the pre-test and post-test.

Regarding the theoretical framework, while attempting to contextualize the research within emotional intelligence and emotional education, we found numerous resources, as it was not difficult to find authors who have worked on both concepts. However, we observed a lack of research focused on the development, management, and understanding of emotions through the teaching-learning process in primary school classrooms.

A potential limitation of the study’s findings might be related to environmental temperature and its effect on emotions. Although the classrooms at the educational center where the study was conducted have air conditioning throughout the school year, the ambient temperature present in the students’ day-to-day could have affected the post-test results. In any case, this variable (environmental temperature) would likely have influenced the rest of the psychosocial and academic variables in a similar manner. Furthermore, it is necessary to point out that the intervention was implemented every 15 days due to school logistical limitations, which precluded the control of other emotion-influencing factors, including sleep, family relationships, peers, and climate. Such constraints are typical of field-based, non-laboratory research.

Currently, there is increasing recognition of the importance of understanding and managing the emotions people experience, as these have secondary effects that impact our well-being, even from an early age (Alarcón-Espinoza et al., 2025; Boobani et al., 2025; Molero et al., 2022).

### 5.2. Prospective

We consider EI to be a sufficiently broad and relevant field, and we do not rule out continued research using the same instruments in future generations. As an improvement to the current work and a proposal for future research, we would like to suggest a similar study that respects the variables examined but with a larger sample. This could be repeated at the same school or a different one, following the same or a very similar procedure. This would allow for the pertinent improvements needed to achieve the established objectives. Among the future lines of action, we consider it useful to analyze the evolution of EI scores during adolescence, as its different components exhibit variation not only due to age-related changes, but also in relation to gender, particularly emotional perception and stress management (Cabello et al., 2016; Candeias, 2025).

Finally, we want to emphasize the importance of making schools welcoming and safe places for students, where they can feel comfortable and be themselves. Unfortunately, many students today feel overwhelmed by everyday situations. The main challenge lies in the difficulty of understanding and managing their emotions, a topic we have addressed in our work. It is crucial for families, friends, teachers, and the entire school environment to take an interest in helping young people develop their EI so they can express themselves with confidence. By doing so, we will foster competent, self-assured, respectful, and empathetic adults.

## Figures and Tables

**Table 1:** Descriptive statistics (means and standard deviations).

	5th Grade (*n* = 74) *M* (*SD*)	6th Grade (*n* = 75) *M* (*SD*)	Total (*n* = 149) *M* (*SD*)
Emotional Awareness and Control (EAC)	9.03 (±2.90)	8.48 (±2.54)	8.70 (±2.73)
Self-esteem (SE)	14.61 (±3.26)	15.42 (±2.85)	15.0 (±3.08)
Social-Emotional Skills (SES)	13.20 (±2.65)	14.19 (±2.30)	13.7 (±2.51)
Life Skills (LS)	13.11 (±2.0)	13.78 (±2.55)	13.5 (±2.31)
Emotional Regulation (ER)	15.23 (±4.81)	15.14 (±4.75)	13.5 (±4.76)

*M:* Mean, *SD*: Standard Deviation.

**Table 2:** Correlation Matrix (Spearman) of the Instrument Dimensions.

	(1) Rho_147_ (p)	(2) Rho_147_ (p)	(3) Rho_147_ (p)	(4) Rho_147_ (p)	(5) Rho_147_ (p)
EAC (1)	-				
SE (2)	−0.491 (*p* < 0.001) ***	-			
SES (3)	−0.062 (*p* = 0.456)	0.193 (*p* = 0.018) *	-		
LS (4)	−0.063 (*p* = 0.450)	0.253 (*p* = 0.002) **	0.357 (*p* < 0.001) ***	-	
ER (5)	−0.381 (*p* < 0.001) ***	0.522 (*p* < 0.001) ***	0.344 (*p* < 0.001) ***	0.306 (*p* < 0.001) ***	-

Note: EAC: Emotional Awareness and Control SE: Self-esteem, SES: Social-Emotional Skills, LS: Life Skills, ER: Emotional Regulation. * *p* < 0.05, ** *p* < 0.01, *** *p* < 0.001.

**Table 3:** Gender differences (boys vs. girls), Mann–Whitney *U* test.

	Boys (*n* = 77) *Mean* (*SD*)	Girls (*n* = 72) *Mean* (*SD*)	Mann–Whitney *U*-test	*p*	Effect Size Hedges’ *g*
EAC	8.53 (±2.85)	8.92 (±2.59)	2434	0.240	0.1115
SE	15.19 (±3.12)	14.83 (±3.03)	2528	0.416	0.0772
SES	13.45 (±2.38)	14.02 (±2.65)	2401	0.193	0.1234
LS	13.45 (±2.45)	13.47 (±2.13)	2651	0.734	0.0323
ER	15.31 (±4.88)	15.09 (±4.64)	2663	0.771	0.0279

Note: EAC: Emotional Awareness and Control, SE: Self-esteem, SES: Social-Emotional Skills, LS: Life Skills, ER: Emotional Regulation. Interpretation Criteria for Effect Size: Hedges’ *g* is interpreted using Cohen’s criteria, where values of 0.2, 0.5, and 0.8 are considered small, medium, and large effect sizes, respectively.

**Table 4:** Differences according to educational level (5th grade vs. 6th grade), Mann–Whitney *U* test.

	5th-Grade (*n* = 74) *Mean* (*SD*)	6th-Grade (*n* = 25) *Mean* (*SD*)	Mann–Whitney *U*-test	*p*	Effect Size Hedges’ *g*
EAC	9.03 (±2.90)	8.42 (±2.54)	2415	0.213	0.11797
SE	14.61 (±3.26)	15.42 (±2.85)	2386	0.174	0.12856
SES	13.20 (±2.65)	14.19 (±2.30)	2086	0.012 *	0.23813
LS	13.11 (±2.00)	13.78 (±2.55)	2366	0.149	0.13605
ER	15.23 (±4.81)	15.14 (±4.75)	2731	0.980	0.00256

Note: EAC: Emotional Awareness and Control SE: Self-esteem, SES: Social-Emotional Skills, LS: Life Skills, ER: Emotional Regulation. *M:* Mean, *SD*: Standard Deviation. * *p* < 0.05. Interpretation Criteria for Effect Size: Hedges’ *g* is interpreted using Cohen’s criteria, where values of 0.2, 0.5, and 0.8 are considered small, medium, and large effect sizes, respectively.

**Table 5:** Group differences (pretest/post-test) and Wilcoxon signed-rank test.

	Experimental (*n* = 25) Pre-Test/Post-Test *Mean* (*SD*)	Control 1 (*n* = 25) Pre-Test/Post-Test *Mean* (*SD*)	Control 2 (*n* = 25) Pre-Test/Post-Test *Mean* (*SD*)
EAC pretest/post-test	7.88 (±2.35)/8.92 (±2.0) * W = 53, *p* = 0.049; *r* = −0.49)	8.20 (±2.18)/9.04 (±1.79) * W = 33, *p* = 0.040; *r* = −0.56)	9.08 (±2.97)/9.68 (±3.0) *W =* 95.5, *p* = 0.319; *r* = −0.24)
SE pretest/post-test	14.08 (±2.58)/14.48(±2.95) *W =* 100.5, *p* = 0.399; *r* = −0.20)	15.92 (±2.86)/15.36 (±2.84) *W =* 122, *p* = 0.279; *r* = 0.28)	16.36 (±2.66)/15.32 (±2.90) * W = 185, *p* = 0.015; *r* = 0.60)
SES pretest/post-test	13.36 (±2.66)/14.24 (±2.13) * W = 56, *p* = 0.038; *r* = −0.51)	14.16 (±2.23)/14.04 (±1.77) *W =* 132, *p* = 0.857; *r* = 0.04)	15.04 (±1.59)/14.40 (±5.89) ** W = 208, *p* = 0.033; *r* = −0.50)
LS pretest/post-test	12.36 (±2.10)/13.32 (±2.17) ** W = 41, *p* = 0.008; *r* = −0.64)	14.12 (±2.55)/14.04 (±1.79) *W =* 106, *p* = 0.516; *r* = −0.15)	14.92 (±2.31)/13.56 (±2.36) ** W = 131.5, *p* = 0.009; *r* = −0.71)
ER pretest/post-test	12.84 (±4.24)/16.00 (±3.89) ** W = 45, *p* = 0.002; *r* = −0.72)	14.36 (±4.74)/15.88 (±4.61) *W =* 71.5, *p* = 0.130; *r* = −0.38)	18.40 (±3.44)/17.20 (±5.94) *W =* 180, *p* = 0.398; *r* = −0.20)

Note: EAC: Emotional Awareness and Control SE: Self-esteem, SES: Social-Emotional Skills, LS: Life Skills, ER: Emotional Regulation. Interpretation Criteria for Effect Size *r*: Interpreted according to Cohen’s criteria, where values of 0.1, 0.3, and 0.5 are considered small, medium, and large effect sizes, respectively. * *p* < 0.05, ** *p* < 0.01.

## Data Availability

Data available in a publicly accessible repository. The original data presented in the study are openly available in FigShare repository at DOI: https://doi.org/10.6084/m9.figshare.30304288.

## Abbreviations

The following abbreviations are used in this manuscript:

EI	Emotional Intelligence
CEE-R	Spanish version of the Emotional Education Questionnaire
WLEIS-S	Wong-Law Emotional Intelligence Scale-Spanish
EAC	Emotional Awareness and Control
SE	Self-esteem
SES	Social-Emotional Skills
LS	Life Skills
ER	Emotional Regulation
